# The Dead Horse: How Its Carcass Is Utilized in the Arts

**Published:** 1896-08

**Authors:** 


**On to Buffalo!**

---

### THE DEAD HORSE: HOW ITS CARCASS IS UTILIZED IN THE MANUFACTURING AND DOMESTIC ARTS.

Horses that have served useful and honorable careers of twenty to thirty years are fit only for the chemical process. When the retired animal is dragged in, it is first relieved of its hair by a shaving process. The tail and mane are especially valuable, and from these is made the haircloth of commerce. The short hair taken from the hide is used for stuffing cushions and horse-collars, and thus the dead are made to minister to the comfort of the living.

The hide of the horse is quite valuable and the leather known as cordovan is made from the skin over the rump. This leather

[1] From the New York World.

is used in the manufacture of high-class hunting and wading boots, and it can be made impervious to water. The other leather is soft and is used mostly for slippers and heavy driving-gloves. The hoofs of the animal are removed, and, after being boiled to extract the oil from them, the horny substance is shipped to the manufacturers of combs and what is known as Mikado goods.

Next the carcass is placed in a cylinder, and cooked by steam at a pressure of three atmospheres. This separates the flesh from the bones. The leg-bones are very hard and white, and are used for handles of pocket and table cutlery. The ribs and head are burned to make boneblack after they have been treated for the glue that is in them. In the calcining of these bones the vapors arising are condensed and form the chief source of carbonate of ammonia, which constitutes the base of nearly all ammoniacal salts. There is an animal oil yielded in the cooking process which is a deadly poison, and enters into the composition of many insecticides and vermifuges.

The bones to make glue are dissolved in muriatic acid, which takes the phosphate of lime away; the soft element retaining the shape of the bone is dissolved in boiling water, cast into squares, and dried on nets. The phosphate of lime, acted upon by sulphuric acid and calcined with carbon, produces phosphorus for lucifer matches. The remaining flesh is distilled to obtain carbonate of ammonia. The resulting mass is pounded up with potash and then mixed with old nails and iron of every description; the whole is calcined and yields little yellow crystals—prussiate of potash, with which tissues are dyed a Prussian blue and iron transformed into steel. It also forms cyanide of potassium and prussic acid, the two most terrible poisons known in chemistry.

In the course of a lawsuit in St. Louis several years ago it was put in evidence that the River Rendering Company, which had the contract for the removal of dead animals from the city streets, made a clear profit of twenty-four dollars on each horse carcass that they handled.

On to Buffalo!

---

Dr. J. S. Cattanach is spending his summer vacation at Newport, R. I.

